# Testing for Non‐Severe Heritable Platelet Function Disorders

**DOI:** 10.1111/ijlh.70192

**Published:** 2026-07-03

**Authors:** Kate Burley, Naomi Cornish, Maria Molina Carretero, Sarah K. Westbury

**Affiliations:** ^1^ Translational Health Sciences, Bristol Medical School University of Bristol Bristol UK; ^2^ Department of Haematology University Hospitals Bristol and Weston NHS Foundation Trust Bristol UK

## Abstract

Heritable platelet function disorders (HPFD) are a diverse group of bleeding disorders characterised by a primary qualitative defect in platelet function rather than platelet number. HPFD may be broadly categorised according to the severity of bleeding, with Glanzmann thrombasthenia and Bernard Soulier syndrome classically considered severe HPFD, with well‐described clinical and laboratory phenotypes. The remaining non‐severe HPFD are less completely characterised and provide additional challenges in diagnosis despite advances in standardising diagnostic approaches for HPFD. Distinguishing patients with non‐severe HPFD from patients who do not have a bleeding disorder may be aided by employing bleeding assessment tools early in patient assessment, although there may be limitations according to patient factors. Distinguishing patients with non‐severe HPFD from those with acquired platelet function disorders and from those with other heritable disorders of haemostasis (including bleeding disorder of unknown cause) requires more sophisticated clinical and laboratory tools, including platelet light transmission aggregometry, secretion and flow cytometry assays, which can categorise many HPFD to the level of the defective platelet pathway and some to the level of an individual protein. The increasing availability of high‐throughput genetic sequencing has made genetic diagnosis possible for some patients with non‐severe HPFD; however, this is limited by difficulties with standardisation of laboratory phenotyping and the challenges of distinguishing pathogenic from benign genetic variation. This review will focus on updates in diagnostic approaches for patients with non‐severe HPFD since the last IJLH education article on this topic in 2014.

## 1 Introduction

Heritable platelet function disorders (HPFD) are disorders of primary haemostasis, including the rare and severe autosomal recessive disorders Glanzmann thrombasthenia (GT) and Bernard Soulier syndrome (BSS) as well as the non‐severe HPFD which are the focus of this review. This diverse group of more common disorders is typically associated with less severe bleeding symptoms than GT or BSS, and results from qualitative defects in platelet functional pathways, including platelet adhesive receptors, receptors for soluble agonists, granule biogenesis and secretion pathways, signalling pathways or platelet procoagulant activity (reviewed recently by Rodriguez‐Alen [1]). These disorders include autosomal dominant and recessive forms, although *de novo* presentations, variable penetrance and environmental confounding may obscure heritability patterns in some families. This review will define non‐severe HPFD as all forms of heritable platelet disorder where there is evidence of a platelet functional defect, regardless of platelet count, but excluding autosomal recessive GT and BSS.

Registry data from UK haemophilia centres [2] suggests a UK population prevalence of approximately 1 in 1 000 000 for GT and BSS and 1 in 40 000 for non‐severe HPFD. New UK haemophilia centre registrations with GT (9 patients) and BSS (14 patients) compared to all other platelet disorders (including heritable disorders of platelet number as well as function, 365 patients) in the 2024–2025 financial year are consistent with this difference in estimated prevalence [2].

The primary objective of diagnostic testing for most patients referred with clinical features suggestive of a non‐severe HPFD is to distinguish those who have the condition from those who do not. This includes identifying those patients who have an alternative heritable cause of bleeding (including, for example, a severe HPFD, von Willebrand disease or a bleeding disorder of unknown cause [BDUC]), those patients with an acquired bleeding disorder and patients with no bleeding disorder. This provides the initial grounds for clinical management of patients at risk of abnormal bleeding. Secondary objectives such as a more precise non‐severe HPFD diagnosis including identification of the defective platelet pathway, protein and gene are achievable for some conditions but may require access to specialist assays and research laboratory testing, which is not feasible for all patients and clinicians. However, a precise diagnosis remains desirable in most patients with non‐severe HPFD whenever possible. Although management of bleeding and prophylaxis during haemostatic challenges is similar and pragmatic for most non‐severe HPFD (with the exception of Quebec platelet disorder), broader management may differ significantly depending on the specific diagnosis. For example, information about inheritance patterns is helpful for genetic counselling of index cases and their families, while some non‐severe HPFD are associated with an increased risk of haematological malignancies, and knowledge of this can inform patient education and follow up. Patients diagnosed with a syndromic form of non‐severe HPFD may benefit from input from other health care teams.

Since the last IJLH educational review on this topic was published [3], national and international guidance for diagnostic strategies and optimised laboratory platelet phenotyping approaches has been updated, and there is much greater access to genetic testing. In this review, we particularly focus on these changes to provide a contemporary account of testing for non‐severe HPFD.

## 2 Identifying and Addressing Variability in Diagnostic Testing Practice

A 2014 worldwide survey involving 202 laboratories across 37 countries found that of ~14 000 patients investigated for a HPFD each year, 40% had a demonstrable platelet function defect and 8.7% of those received a molecular diagnosis [4]. The same study reported that the most widely used tests for diagnosing suspected HPFDs included light transmission aggregometry (LTA; 59% of laboratories) and the Platelet‐Function Analyser‐100 (PFA‐100) closure time assay (58% of laboratories). Most laboratories also performed a complete blood count and peripheral blood smear; however, only 30% of centres used a standardised bleeding assessment tool (BAT) to evaluate bleeding severity prior to diagnostic testing. A subsequent survey of Northern European diagnostic coagulation centres [5] found that although aggregometry was routinely used to evaluate for HPFD in all laboratories, there was substantial variation in the preferred method, with approximately 35% of laboratories performing whole blood Multiplate aggregometry for assessment of HPFD. Many laboratories (71%) also performed flow cytometry for selected patients with suspected HPFD while molecular genetic testing was only available in half of the centres surveyed.

To try to improve the consistency of approach, the International Society on Thrombosis and Haemostasis (ISTH) Platelet Physiology SSC published consensus guidance in 2015 outlining diagnostic approaches to HPFD. This recommended use of LTA, platelet secretion assays (PSA) and flow cytometry as first‐line diagnostic tests, following initial clinical and laboratory screening, as summarised in Figure 1 [6]. PFA‐100 assays and bleeding times were not recommended due to inadequate sensitivity and specificity, and poor reproducibility. Second‐ and third‐line diagnostic tests were recommended for patients who did not achieve a diagnosis in the initial phase of investigations; however, many of these tests were highly specialised tests most commonly available in research settings rather than clinical laboratories [6]. This document remains the most internationally placed guidance for diagnosis of HPFD. Further guidance from the ISTH Platelet Physiology SSC has been published regarding the application of flow cytometry [7] and PSA [8] in the diagnosis of platelet disorders including HPFD. Subsequently, several national and regional guidelines have been published that potentially offer a more nuanced approach reflecting local disease prevalence and availability of specialised testing [9, 10, 11, 12, 13].

**FIGURE 1:**
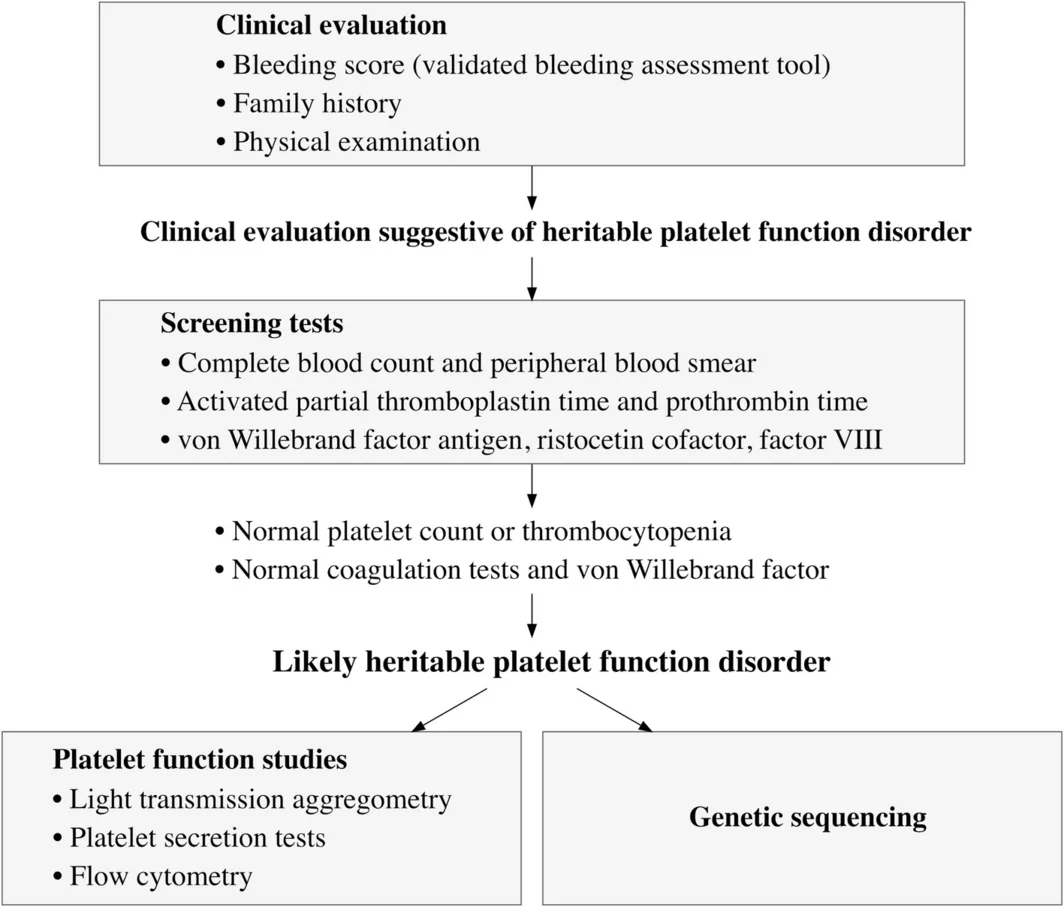
Approach to testing for HPFDs based on the 2015 ISTH Platelet Physiology SSC guidance [6].

## 3 Clinical Evaluation and Screening Investigations

Clinical evaluation should involve a detailed bleeding history, supported by a validated BAT [6]. Haemorrhagic symptoms may be more apparent in women due to menstruation and childbirth [14]. A large validation study of the ISTH‐BAT demonstrated that a score above 4 (in women) and 3 (in men) reliably discriminated between patients with HPFD and healthy controls, providing von Willebrand disease had been excluded [15]. However, in patients with non‐severe HPFDs, the ISTH‐BAT may be low and decisions to pursue further investigation should include careful consideration of other factors, including family history and extra‐haematological features [16]. Physical examination may identify syndromic features which could be associated with an HPFD, such as oculocutaneous hypopigmentation in Hermansky–Pudlak‐type syndromes, which may prompt more targeted evaluation. Acquired causes of platelet dysfunction, including drugs, food supplements, renal failure or myeloid malignancy should also be excluded [10].

For patients where clinical assessment is suggestive of a possible HPFD, initial laboratory screening includes a complete blood count, coagulation tests and von Willebrand disease screen (to rule out alternative bleeding conditions) and examination of peripheral blood morphology using light microscopy [6]. Abnormalities in platelet number, size and granularity, or changes in other blood cell number or morphology may provide clues to the underlying diagnosis. For example, thrombocytopenia with platelets of normal size and granulation could be consistent with HPFD associated with variants in RUNX1, ANKRD26 or ETV6, while macrothrombocytes with reduced alpha granulation may be suggestive of grey platelet syndrome. Morphological abnormalities of other blood cells may help identify HPFD, for example red cell features suggestive of GATA1‐associated disorders, or leucocyte abnormalities in Chediak‐Higashi syndrome [17]. Unfortunately, many non‐severe HPFD are not associated with a distinctive blood film appearance by conventional light microscopy. Indirect immunofluorescence analysis of blood smears [18] may provide additional information but is not currently widely available or integrated into clinical diagnostic algorithms.

## 4 Laboratory Phenotyping Tests for Non‐Severe HPFD

### 4.1 Light Transmission Aggregometry (LTA)

For patients with a normal coagulation screen and normal platelet count or mild thrombocytopenia, first‐line testing for HPFD should include LTA performed in platelet‐rich plasma (PRP). This remains the reference laboratory method for assessing platelet aggregation and provides the most comprehensive functional evaluation of primary platelet activation pathways [19, 20]. Traditionally, extended agonist panels at multiple concentrations have been used to interrogate receptor‐mediated pathways, intracellular signalling, and amplification mechanisms involving thromboxane A_2_ and granule secretion. Although analytically sensitive across a broad spectrum of platelet defects, this approach has historically been labour‐intensive and susceptible to considerable pre‐analytical and analytical variability [21, 22] despite several international working groups producing detailed methodological guidance covering pre‐analytical variables, blood collection and handling, PRP preparation, agonist selection, and interpretation of aggregation traces [23, 24, 25].

To improve diagnostic efficiency and inter‐laboratory comparability, current ISTH and British Society for Haematology (BSH) guidelines recommend a tiered approach to agonist selection [6, 10]. A defined core agonist panel is advised for initial assessment, with targeted expansion using an extended panel depending on clinical phenotype or preliminary LTA results. Table 1 summarises the recommended initial and full agonist panels, together with typical working concentrations, as outlined in the current British Society for Haematology (BSH) [10, 23, 26]. Adoption of a structured approach supports harmonisation between laboratories and ensures adequate diagnostic coverage for the most common defects implicated in non‐severe HPFD. However, the limited panel may not be sensitive to all non‐severe HPFD, will not allow parallel assessment of responses to arachidonic acid and the thromboxane analogue U46619, and may necessitate repeat testing for some patients. For this reason, we prefer to include U46619 alongside the initial panel whenever possible. The BSH guidelines are specifically tailored to situations where analyser channels are limited, or where there are limitations due to small sample volumes, in which case the emphasis is placed on ruling out a severe HPFD [10].

**TABLE 1:** Recommended LTA agonist panel for suspected heritable platelet disorders.

Agonist	Concentration (initial panel)	Concentration (full panel)	Receptor(s)	Mechanism/pathway activated
ADP	5 and 10 μM	5 and 10 μM	P2Y_1_, P2Y_12_	P2Y_1_ → transient shape change; P2Y_12_ (Gi) → amplification of aggregation + secretion/granule release, supports secondary wave.
Collagen (type I, tendon)	1 or 1.25 μg/mL	1 or 1.25 μg/mL and 2 or 2.50 μg/mL	GPVI (primary), integrin α_2_β_1_	ITAM mediated activation → strong platelet activation, granule secretion, thromboxane A_2_ generation; tests collagen receptor/signalling integrity.
Epinephrine	5 and 10 μM	5 and 10 μM	α_2_ adrenergic receptor	Weak agonist—relies on secretion and amplification (Gi pathway); useful to detect secretion or Gi pathway defects, granule release abnormalities.
Arachidonic acid	1 mM	1 mM	Indirect: COX1 → thromboxane A_2_ → TP receptor	Tests cyclo‐oxygenase (COX1) → TXA_2_ generation and downstream platelet activation; useful for detecting aspirin‐like defects or thromboxane pathway abnormalities.
Ristocetin	0.5 and 1.25 g/L	0.5 and 1.25 g/L	GPIb–IX–V (via von Willebrand factor)	Assesses VWF–GPIb interaction → platelet agglutination; standard dose for GPIbIXV defects or VWD type 1/2, low‐dose for gain‐of‐function VWF/platelet type VWD (hyper‐agglutination).
TRAP6 (thrombin receptor agonist peptide)	—	5 and 10 μM	PAR1 (thrombin receptor)	Activates thrombin signalling via PAR1 → strong Gq/G12/13 signalling, robust platelet activation and secretion; helps identify signalling/secretion defects not apparent with ADP/collagen.
U46619 (thromboxane A_2_ analogue)	—	1 μM	TP (thromboxane) receptor	Bypasses COX1 to directly stimulate TP receptor → tests thromboxane receptor signalling and downstream aggregation/secretion, useful when AA response is inconclusive or for confirmation of thromboxane pathway defects.
NaCl (saline)	—	0.9% w/v	—	Blank/baseline—ensures that aggregation/secretion responses are stimulus‐dependent and provides calibration for maximum light transmission (0% aggregation baseline).

*Note:* This recommendation is based on published BSH guidance [10]. It is noted that the initial panel has limitations (e.g., in ability to detect aspirin‐like defects) but is designed to demonstrate normal platelet aggregometry responses in routine clinical practice, particularly when an eight‐channel analyser is used.

Further methodological refinement has focused on the influence of platelet count in PRP. Some laboratories dilute PRP with autologous platelet‐poor plasma (PPP) to achieve standardised platelet concentrations; however, dilution can artefactually reduce aggregation responses, particularly to weak agonists such as ADP, epinephrine, or collagen. For non‐thrombocytopenic samples, one study has shown only small differences in performance of platelet count adjusted PRP and native PRP in detecting platelet function disorders, with marginal superiority of adjusted PRP in this setting [27]. Current guidance recommends analysing native PRP whenever possible, reserving dilution only for cases of extreme thrombocytosis [10, 27, 28, 29].

A frequent concern relates to the use of LTA in the setting of thrombocytopenia or where PRP has a lower‐than‐expected platelet count despite an adequate platelet count in peripheral blood. These scenarios are not the same, but both may result in problems interpreting the results of LTA, where reduced platelet concentrations may impair platelet aggregometry responses independently of defects in platelet function. Approaches to this have included prioritising other laboratory approaches such as flow cytometry or molecular genetic testing, use of gravity sedimentation to improve platelet recovery in macrothrombocytopenia, adjusting agonist panels to omit weak agonists, and constructing reference ranges based on control samples diluted with autologous PPP [10, 30, 31]. A recent consensus paper from the ISTH has highlighted the challenges in this area [32]. Caution is advised when using LTA to assess platelet function in patients with thrombocytopenia and a platelet count below 75 × 10^9^/L. LTA may still be informative in excluding some specific severe HPFD with platelet counts below this threshold, with the use of adjusted reference ranges necessary for assessment for non‐severe HPFD. This does not avoid the requirement for a native control PRP sample run in parallel to the patient sample to confirm the assay is performing as expected, without issues related to reagents or the analyser [32].

Automated LTA platforms aim to overcome key limitations of manual LTA by reducing operator dependence, improving standardisation, and simplifying workflows, thereby facilitating broader use in routine laboratories [33, 34]. Recent evaluations of dedicated automated systems such as the Thrombomate XRA (TXRA) demonstrate high analytical precision for maximum aggregation (CV approximately 1.4%–4.6%) and good agreement with conventional manual LTA, supporting their analytical reliability [34]. The ability to perform testing using PRP alone, with optional automated or ‘virtual’ platelet‐poor plasma calibration, represents an important advance in assay standardisation and efficiency [34]. Automated LTA implemented on routine coagulation analysers (e.g., Sysmex CS‐ and CN‐Series) also shows reproducible aggregation responses and strong correlation with traditional methods, enabling integration of platelet aggregation testing into automated laboratory workflows [33, 35]. Despite these advances, the continued need for expert interpretation of complex aggregation curves remains an important limitation of automated LTA systems [33, 34].

Expansion of external quality‐assessment (EQA) schemes has also contributed to improved standardisation. Technical challenges due to the short time window between sampling and analysis previously restricted the availability of high‐quality abnormal reference samples for assessment [36], and EQA schemes were often limited to interpretation of externally generated LTA traces, such as those provided by the North American Specialized Coagulation Laboratory Association [37]. However, a recent UK NEQAS pilot distributing cold‐stored donor platelets demonstrated high interlaboratory concordance for classical agonists (ADP, AA, collagen, epinephrine, and ristocetin), confirming the feasibility of harmonised LTA performance across multiple centres [38]. Ongoing development of such programmes is expected to further enhance reproducibility and promote broader adoption of standardised LTA protocols.

### 4.2 Platelet Secretion Tests

Platelet secretion is a central amplification mechanism of platelet activation and is essential for sustained aggregation and thrombus stabilisation. Defects in granule secretion are a common finding in patients with non‐severe HPFD. Expert consensus and laboratory practice surveys emphasise that secretion defects are frequently underdiagnosed if platelet aggregation is used in isolation, underscoring the need for dedicated secretion testing [5, 8, 39]. However, these tests are highly specialised, are performed in limited numbers of laboratories and can be associated with significant preanalytical, analytical and postanalytical challenges. Furthermore, ATP secretion results can be highly variable in control samples, and one study has shown that ATP secretion results do not predict an elevated bleeding score or diagnosis of a bleeding disorder [40]. Expert recommendations and recent reviews still suggest that impaired ATP release in response to multiple agonists is a hallmark of secretion defects and provides clinically meaningful information beyond aggregation responses alone [8, 39]. These assays may be valuable for distinguishing secretion defects from signalling abnormalities and primary aggregation defects in non‐severe HPFD, but this is an area where further work to improve assay reliability is needed.

Alpha‐granule secretion is typically assessed by flow cytometric measurement of surface P‐selectin (CD62P) expression following platelet activation. Standardised flow cytometry–based assays are increasingly recognised as essential tools for identifying α‐granule secretion defects and combined granule disorders [8, 41]. Simplified and reproducible flow cytometric protocols have improved inter‐laboratory feasibility and sensitivity, supporting their integration into routine diagnostic workflows [5, 41]. Evaluation of both dense and alpha granule secretion is recommended to capture the full spectrum of platelet secretory abnormalities [8].

Table 2 summarises the principal assays used to assess platelet secretion, the granule populations evaluated, and their diagnostic utility. Together, quantitative assessment of dense granule content, ATP release assays, and flow cytometric analysis of α‐granule secretion provide a comprehensive and complementary framework for detecting platelet secretion defects and for distinguishing storage pool deficiencies from release defects in patients with suspected non‐severe HPFD [5, 8, 39].

**TABLE 2:** Summary of platelet secretion assays and their diagnostic utility.

Assay category	Method	What it measures	Diagnostic utility	Strengths	Limitations
Dense granule content assays	ATP/ADP quantification (bioluminescence or HPLC)	Total intracellular ADP and ATP (δ‐granule content)	Identification of δ‐storage pool deficiency	Sensitive; quantitative; reproducible	Does not assess release function
Dense granule secretion assays	ATP luminescence (Lumi‐aggregometry)	Agonist‐induced ATP release from δ‐granules	Detection of secretion (release) defects; complements LTA	Simultaneous aggregation + secretion; kinetic data	Requires PRP; relatively large sample volume; influenced by platelet count
Alpha‐granule secretion assays	Flow cytometric detection of P‐selectin (CD62P)	Surface expression following activation	Identification of α‐granule secretion defects and activation abnormalities	Whole‐blood compatible; small sample volume; suitable for paediatric testing	Requires standardised activation conditions
Combined secretion and activation markers	Flow cytometry panels (e.g., CD62P + integrin activation markers)	Multiple functional outputs per stimulus	Broader assessment of secretory and activation pathways	Whole‐blood compatible; small sample volume; suitable for paediatric testing	Requires technical expertise; inter‐lab variability

### 4.3 Flow Cytometric Assessment of Platelets Beyond Secretion Assays

Flow cytometry of resting platelets to detect expression of GPIIb/IIIa (integrin α_IIb_β_3,_ CD41/CD61), GPIb (CD42b) and GPIb/IX (CD42a) and of activated platelets to detect a GPIIb/IIIa activation epitope (PAC‐1) is recommended as a first step diagnostic test in patients with suspected HPFD, predominantly to identify those patients with the severe HPFD GT or Bernard Soulier syndrome [6]. In the same guideline, extended flow cytometric panels to evaluate the glycoproteins GPIa/IIa (CD31 and CD49b), GPIV (CD36) and GPVI (to identify rarer platelet glycoprotein abnormalities) as well as assessment of platelet procoagulant activity by quantification of annexin V binding (to detect Stormorken and Scott syndrome) were proposed as second step diagnostic tests for HPFD in patients who did not achieve a precise diagnosis during the first round of investigations. Analysis of platelet surface glycoproteins with flow cytometry remains recommended in selected patients in updated guidance for patients with suspected HPFD [7], including patients with thrombocytopenia [42].

## 5 Molecular Diagnostic Testing

The catalogue of genes involved in megakaryopoiesis, platelet biogenesis, platelet function, and heritable bleeding phenotypes has expanded substantially over the past decade, largely driven by the introduction of high‐throughput sequencing (HTS) technologies in research settings. At least 68 genes are robustly linked to inherited platelet number and function disorders, with some evidence of abnormal platelet function associated with variants in the majority of these genes [43]. As next‐generation sequencing (NGS) platforms have become more affordable, accurate, and computationally accessible, these approaches have been increasingly adopted into clinical practice, markedly improving diagnostic yield for patients with non‐severe HPFDs [44, 45, 46]. Improvements in genome curation, variant annotation resources, and population reference datasets (e.g., gnomAD) have further strengthened variant interpretation, enabling genomics to become an integral component of the standard diagnostic pathway [47].

### 5.1 Targeted Approaches and Sanger Sequencing

In some patients, a well‐defined clinical and laboratory phenotype may clearly implicate a specific protein defect, allowing selection of a single candidate gene for Sanger sequencing. Examples include the characteristic blood film features of Gray Platelet Syndrome (*NBEAL2*) or the syndromic features associated with Hermansky‐Pudlak syndrome. Targeted Sanger sequencing is also appropriate when a familial pathogenic variant has already been identified, enabling rapid and cost‐effective cascade testing. However, sequential analysis of multiple individual genes becomes impractical and costly when the phenotype is non‐specific or when numerous plausible candidate genes exist.

### 5.2 Use of NGS Gene Panels

For most patients with confirmed platelet dysfunction but non‐specific or overlapping clinical and laboratory features, the preferred diagnostic strategy is an NGS gene panel, particularly when laboratory testing (e.g., light transmission aggregometry or flow cytometry) does not provide sufficient discriminatory power [6]. Targeted panels allow simultaneous evaluation of numerous genes implicated in HPFDs, reducing time to diagnosis and overall cost relative to stepwise Sanger sequencing. Many clinically used panels are based on, or informed by, the International Society on Thrombosis and Haemostasis (ISTH) Scientific and Standardization Committee (SSC) on Genomics in Thrombosis and Haemostasis gene curation framework, which assigns *Tier 1* (‘gold standard’) status to genes with strong evidence for disease causation [43]. Evidence supporting Tier 1 designation typically includes the identification of pathogenic variants in three or more unrelated pedigrees with consistent phenotypes, robust co‐segregation data, and supportive functional evidence, ideally including in vivo modelling [48].

Early targeted sequencing initiatives, notably the ThromboGenomics project and the UK Genotyping and Phenotyping of Platelets (UK‐GAPP) consortium, demonstrated the clinical utility of this approach. In ThromboGenomics' large cohort of patients with suspected heritable platelet disorders, HTS identified pathogenic or likely pathogenic variants in approximately 30%–50% of cases overall, confirming the value of comprehensive targeted testing [46, 49]. However, diagnostic yield was not uniform across phenotypic groups: yields were consistently higher in heritable thrombocytopenias and lower in isolated platelet function disorders, reflecting both greater genetic heterogeneity and incomplete knowledge of the genes underpinning functional defects. Similar patterns were observed in UK‐GAPP, where the likelihood of achieving a molecular diagnosis was significantly greater in patients with well‐defined syndromic or thrombocytopenic presentations than in those with non‐syndromic functional defects [44, 50]. Limitations of panel‐based testing for platelet function disorders underscore the need for continued gene discovery and improved functional annotation.

### 5.3 Variant Interpretation and Reporting

Interpretation of candidate variants requires careful appraisal of multiple lines of evidence, including allele frequency (typically very low for deleterious platelet genes given strong negative selection), predicted functional impact, comparison with disease‐specific variant databases, and segregation analysis where possible [49]. Particular caution is required when evaluating variants of uncertain significance (VUS), which remain common in hereditary platelet disorders due to incomplete gene‐phenotype correlations and limited functional data for many genes. To mitigate these challenges, multidisciplinary team (MDT) review is widely recommended, bringing together clinical haematologists, clinical scientists, geneticists, and, where applicable, laboratory platelet experts. MDT discussion provides a structured framework to integrate molecular findings with clinical phenotype, family history, and laboratory results, helping to avoid overinterpretation of inconclusive variants.

In cases where a VUS may be contributory, additional information can be invaluable. This includes segregation studies within the family, reassessment of the phenotype in relatives, review of historical platelet investigations, or targeted functional assays where feasible. Periodic re‐evaluation of VUS in light of emerging evidence, updated population allele frequency data, or newly published disease associations is also an important component of good genomic practice. Together, these approaches help ensure that reported variants are interpreted cautiously, consistently, and in a clinically meaningful manner.

Ethical considerations may also arise. Variants in some platelet disorder genes, including *ANKRD26*, *ETV6*, and *RUNX1*, are associated not only with thrombocytopenia and platelet dysfunction but also with an increased risk of myeloid malignancies [51]. This necessitates appropriate informed consent, clear communication of potential implications, and structured haematological surveillance when such variants are identified.

## 6 Future Directions

Although targeted NGS gene panels currently represent the standard for clinical diagnostics, whole‐exome sequencing (WES) and whole‐genome sequencing (WGS) are increasingly considered in unresolved cases. WES provides broad coverage of coding regions, enabling detection of variants in both established and emerging heritable platelet disorder genes, whereas WGS offers more uniform coverage and captures the full spectrum of genomic variation, including intronic, regulatory, and structural variants often missed by panel‐based approaches [52]. Importantly, large‐scale national initiatives such as the UK 100000 Genomes Project have demonstrated the feasibility and clinical value of incorporating WGS into routine rare disease diagnostics, including haematological disorders, and have paved the way for broader implementation within genomic medicine services. As sequencing costs decline and analytic capabilities expand, WGS is likely to become the first‐line genomic test for many patients with suspected HPFDs.

Despite these advances, a substantial proportion of patients still lack a molecular diagnosis, underscoring the presence of undiscovered genes, pathogenic mechanisms beyond protein‐coding regions, and abnormalities not detectable by short‐read sequencing alone. Integrative approaches—incorporating RNA sequencing, long‐read sequencing, and single‐cell transcriptomics—are increasingly applied in research settings to address these gaps. RNA‐seq enables detection of cryptic splice defects and altered gene expression; long‐read technologies improve identification of complex structural variants and full‐length isoforms; and single‐cell approaches resolve megakaryocyte differentiation pathways at high resolution. Collectively, these tools enhance interpretation of VUS, refine mechanistic understanding, and support discovery of novel genetic contributors to platelet disorders. As analytic frameworks mature, such multi‐omic strategies are likely to complement WGS and contribute to a more comprehensive diagnostic approach for HFPD.

As access to genomic testing continues to expand, the question is raised about the relative contributions of phenotypic versus genotypic laboratory testing, and the order in which these tests should be performed. In many clinical settings, it may be easier to arrange genomic testing than specialised platelet testing. Therefore, even though genomic testing remains low yield for non‐severe HFPD compared to platelet functional testing [53], there might be an argument for placing this earlier in the diagnostic approach in some settings. For many patients, interpretation of the results of genomic testing may still require additional platelet testing to confirm or refute the suggested molecular diagnosis. Improving access to these phenotypic tests is important to ensure improved diagnostic accuracy across different health care settings.

One technique that shows promise in this regard is use of indirect immunofluorescence microscopy, which has shown promise in identifying different forms of HPFD [18, 54]. It is not currently widely available and has so far been applied to a small number of non‐severe HPFD. However, it has the potential to be used in a wide range of health care settings, with the possibility to send away blood smears for centralised analysis. This technique has the potential to be used early in the diagnostic pathway, especially for centres without easy access to genetic testing, or later, to assist with interpretation of genetic results. Further work to develop and validate this technique for a range of non‐severe HPFD would be valuable, as well as potentially automating analysis. Developing guidance for the diagnosis of non‐severe HPFD in the emerging era of ‘genomics first’ diagnostic pathways will be a challenge for the international platelet community in the next decade.

## Funding

K.B. is supported by an NIHR Academic Clinical Lectureship. N.C. is supported by the Wellcome Trust: grant number 225541/Z/22/Z (GW4‐CAT‐HP doctoral fellowship).

## Conflicts of Interest

The authors declare no conflicts of interest.

## Data Availability

Data sharing not applicable to this article as no datasets were generated or analysed during the current study.
